# Supplementary material for: ProteinShader: illustrative rendering of macromolecules
Source: BMC Struct Biol. 2009 Mar 30;9:19. doi: 10.1186/1472-6807-9-19 (PMC2672931; doi:10.1186/1472-6807-9-19)
Supplement: Additional file 1 — ProteinShader program without source code. This compressed file contains the complete ProteinShader program including associated libraries, but no source code. A README.txt file gives an overview of the ProteinShader distribution, and the index.html file in the help subdirectory has directions on getting started with the program as well as a set of tutorials. [file 1472-6807-9-19-S1.zip › ProteinShader-beta-0_9_4-binary/help/api/org/proteinshader/graphics/Shape.html]

Shape (ProteinShader API)


|  |  |  |
| --- | --- | --- |
| **Overview** \| **Package** \| **Class** \| **Use** \| **Tree** \| **Deprecated** \| **Index** \| **Help** | |  |
| **PREV CLASS**   **NEXT CLASS** | **FRAMES**    **NO FRAMES**     **All Classes** |  |
| SUMMARY: NESTED \| FIELD \| CONSTR \| METHOD | DETAIL: FIELD \| CONSTR \| METHOD |  |


---


## org.proteinshader.graphics Class Shape

```
java.lang.Object
  org.proteinshader.graphics.Shape
```

**Direct Known Subclasses:**: Cylinder, ExtrudedShape, Sphere

---

``` public abstract class Shape extends Object ```

Contains common methods needed by classes that draw geometric objects.

---

| **Field Summary** | |
| --- | --- |
| `static double` | `HALF_PI`             One-half Pi is defined as 1.57079632679489. |
| `static double` | `PI`             Pi is defined as 3.14159265358979. |
| `static float[]` | `SPECULAR_COLOR`             The material specular color is (0.95, 0.95, 0.95, 1.0). |
| `static double` | `TWO_PI`             2\*Pi is defined as 6.28318530717958. |


**Constructor Summary**

`Shape()`
  
          Constructor for use by subclasses.


| **Method Summary** | |
| --- | --- |
| `void` | `deleteDisplayList(GL gl, int displayListName)`             Deletes an OpenGL display list. |
| `void` | `executeDisplayList(GL gl, double scale, int displayListName)`             Executes an OpenGL display list and uniformly scales the geometric object. |
| `void` | `executeDisplayList(GL gl, int displayListName)`             Executes an OpenGL display list. |
| `void` | `setMaterial(GL gl, float red, float green, float blue, float alpha, float specularExp)`             Sets the material properties for the object about to be drawn. |

**Methods inherited from class java.lang.Object**

`clone, equals, finalize, getClass, hashCode, notify, notifyAll, toString, wait, wait, wait`

**Field Detail**

### PI

```
public static final double PI
```

:   Pi is defined as 3.14159265358979.

    **See Also:**: Constant Field Values

---


### TWO\_PI

```
public static final double TWO_PI
```

:   2\*Pi is defined as 6.28318530717958.

    **See Also:**: Constant Field Values

---


### HALF\_PI

```
public static final double HALF_PI
```

:   One-half Pi is defined as 1.57079632679489.

    **See Also:**: Constant Field Values

---


### SPECULAR\_COLOR

```
public static final float[] SPECULAR_COLOR
```

:   The material specular color is (0.95, 0.95, 0.95, 1.0).


**Constructor Detail**

### Shape

```
public Shape()
```

:   Constructor for use by subclasses.


**Method Detail**

### executeDisplayList

```
public void executeDisplayList(GL gl,
                               double scale,
                               int displayListName)
```

:   Executes an OpenGL display list and uniformly scales the geometric
    object.

    :   **Parameters:**: `gl` - the current GL object.: `scale` - the scale factor for the sphere.: `displayListName` - the name (an integer) of an OpenGL display list.

---


### executeDisplayList

```
public void executeDisplayList(GL gl,
                               int displayListName)
```

:   Executes an OpenGL display list.

    :   **Parameters:**: `gl` - the current GL object.: `displayListName` - the name (an integer) of an OpenGL display list.

---


### deleteDisplayList

```
public void deleteDisplayList(GL gl,
                              int displayListName)
```

:   Deletes an OpenGL display list. If a display list is no longer
    needed, its memory on the graphics card should be freed up by
    calling this method. If the name given as an argument does not
    correspond to an existing display list, it is simply ignored.

    :   **Parameters:**: `displayListName` - the name (an integer) of an OpenGL display list.

---


### setMaterial

```
public void setMaterial(GL gl,
                        float red,
                        float green,
                        float blue,
                        float alpha,
                        float specularExp)
```

:   Sets the material properties for the object about to be drawn.


---


|  |  |  |
| --- | --- | --- |
| **Overview** \| **Package** \| **Class** \| **Use** \| **Tree** \| **Deprecated** \| **Index** \| **Help** | |  |
| **PREV CLASS**   **NEXT CLASS** | **FRAMES**    **NO FRAMES**     **All Classes** |  |
| SUMMARY: NESTED \| FIELD \| CONSTR \| METHOD | DETAIL: FIELD \| CONSTR \| METHOD |  |


---

# *Copyright © 2007-2008*
